# Evaluation of Peripheral Blood and Cord Blood Platelet Lysates in Isolation and Expansion of Multipotent Mesenchymal Stromal Cells

**DOI:** 10.3390/bioengineering5010019

**Published:** 2018-02-26

**Authors:** Ioanna Christou, Panagiotis Mallis, Efstathios Michalopoulos, Theofanis Chatzistamatiou, George Mermelekas, Jerome Zoidakis, Antonia Vlahou, Catherine Stavropoulos-Giokas

**Affiliations:** 1 Hellenic Cord Blood Bank, Biomedical Research Foundation Academy of Athens, 4 Soranou Ephessiou Street, 115 27 Athens, Greece; x.janna@hotmail.com (I.C.); pmallis@bioacademy.gr (P.M.); tchatzistamatiou@dha.gov.ae (T.C.); cstavrop@bioacademy.gr (C.S.-G.); 2 Biotechnology division, Biomedical Research Foundation Academy of Athens, 4 Soranou Ephessiou Street, 115 27 Athens, Greece; gmermelekas@yahoo.com (G.M.); izoidakis@bioacademy.gr (J.Z.); vlahoua@bioacademy.gr (A.V.)

**Keywords:** Cord blood, Multiple Reaction Monitoring, multipotent Mesenchymal Stem Cells, peripheral blood, platelet lysate

## Abstract

**Background:** Multipotent Mesenchymal Stromal Cells (MSCs) are used in tissue engineering and regenerative medicine. The in vitro isolation and expansion of MSCs involve the use of foetal bovine serum (FBS). However, many concerns have been raised regarding the safety of this product. In this study, alternative additives derived either from peripheral or cord blood were tested as an FBS replacement. **Methods:** Platelet lysates (PL) from peripheral and cord blood were used for the expansion of MSCs. The levels of growth factors in peripheral blood (PB) and cord blood (CB) PLs were determined using the Multiple Reaction Monitoring (MRM). Finally, the cell doubling time (CDT), tri-lineage differentiation and phenotypic characterization of the MSCs expanded with FBS and PLs were determined. **Results:** MSCs treated with culture media containing FBS and PB-PL, were successfully isolated and expanded, whereas MSCs treated with CB-PL could not be maintained in culture. Furthermore, the MRM analysis yielded differences in growth factor levels between PB-PL and CB-PL. In addition, the MSCs were successfully expanded with FBS and PB-PL and exhibited tri-lineage differentiation and stable phenotypic characteristics. **Conclusion:** PB-PL could be used as an alternative additive for the production of MSCs culture medium applied to xenogeneic-free expansion and maintenance of MSCs in large scale clinical studies.

## 1. Introduction

The field of tissue engineering and regenerative medicine is rapidly evolving and involves the use of specified and unspecified cellular populations in combination with various types of scaffolds [1,2]. Currently, multipotent Mesenchymal Stromal Cells (MSCs) are clinically used in approaches of regenerative medicine and cellular therapies [3,4,5]. However, these applications demand a significant number of in vitro expanded MSCs [6,7]. Common culture methods for the expansion of MSCs involve the use of foetal bovine serum (FBS) as a supplement, in combination with basal culture medium [8,9]. A satisfactory number of these cells can be obtained easily from different sources including bone marrow, umbilical cords, Wharton jelly, umbilical cord blood, amniotic fluid and lipoaspirates from adipose tissue [10,11,12,13]. According to the International Society for Cellular Therapy (ISCT), MSCs are defined as plastic adherent cells, positive for specific surface antigens including CD105, CD73, CD90 and negative for hematopoietic markers such as CD45, CD34, CD14, CD19 and HLA class II and trilineage mesodermal differentiation to adipocytes, chondroblasts and osteoblasts [14,15,16,17]. Recently, it was determined that the source of MSCs affects significantly their characteristics such as their heterogeneity in morphology, proliferative activity, differentiation and therapeutic potentials [17,18].

Furthermore, MSCs have the ability to secrete a variety of trophic factors that contribute to tissue remodelling and immunomodulation and can be applied as first or second line treatment for various diseases [19,20,21].Moreover, combining them with chitosan scaffolds, could be a useful tool for osteochondral tissue regeneration [22,23].Additionally, due to their immunomodulatory properties, MSCs represent an attractive cell source for treatment of autoimmune disorders such as multiple sclerosis (MS), amyotrophic lateral sclerosis (ALS), type I diabetes mellitus, Crohn’s disease and systemic lupus erythematosus [24,25,26,27]. It has been proven that MSCs can adjust the immunoreaction directly or indirectly [28]. In a direct manner, MSCs can induce apoptosis of T cells through Fas/Fas ligand and TNF receptor signalling pathways. Alternatively, apoptosis in T cells can be induced via the secretion of IL-6, IL-10, nitric oxide (NO), idoleamine 2,3 dioxygenase (IDO) and prostaglandin E_2_ (PGE_2_). In this way, the autoreactive T cell population can be adjusted, thus providing enough time to the damaged tissues to remodel and regenerate [28]. Currently, clinical trials using MSCs for ischemic stroke, myocardial infraction and graft versus host disease have also been performed worldwide [29,30].

In order to support, the wide use of MSCs in regenerative medicine and tissue engineering approaches, in vitro culturing and expansion conditions must be developed. In addition, large scale clinical translation trials in accordance with good manufacturing practices (GMP) requires the use of a well-defined culture medium in order to maintain the cellular quality, while avoiding adverse patient reactions [30]. Nowadays, FBS, derived from the whole blood of bovine foetuses, is the most widely used supplement for cell culture medium preparation. FBS is a rich source of growth factors like transforming growth factor- beta 1 (TGF-β1), fibroblast growth factor (FGF), epidermal growth factor (EGF), vascular endothelial growth factor (VEGF), platelet derived growth factor (PDGF), insulin-like growth factor (IGF), growth hormones and albumin. Thus, it is the optimum additive in culture for the expansion of various types of cells [31,32]. However, many concerns are arising regarding the safety of this product are arising. FBS could contain prions (causing mad cow disease), xenogeneic antigens, bovine proteins or transfer zoonotic infections to the cultured cells. These cause significant complications to patients receiving cultured MSC therapies [33,34]. Another disadvantage is the different concentration in the amount of growth factors between different lots of FBS. Annually, it is estimated that 600,000 L of FBS are demanded for cell culturing but only 1/3 is suitable for GMP use and clinical grade cell expansion. However, more than 200 phase I/II clinical trials, report the use of FBS as the primary supplement for the in vitro expansion of MSCs (according to www.clinicaltrials.gov as of 25 March 2013).

To address these issues, alternative strategies for the culture and expansion of cells are currently being developed and focused in the production of culture medium free of any animal derivatives. The substitution of FBS with human serum has provided contradictory results in the expansion, proliferation and differentiation capacities of MSCs [35,36,37]. Human platelet lysate (hPL) from pooled expired plasma apheresis showed promising results, when used in MSC culture. Given that, hPL contains significant amounts of TGF-β1, FGF, VEGF, PDGF and IGF it can be used efficiently for MSC applications [38,39]. One serious drawback regarding the use of hPL is the availability of expired pooled plasma apheresis. Umbilical cord blood, could possibly address this problem, thus providing an alternative source for the production of platelet lysate, since it contains a similar number of platelets with the peripheral blood. In addition, a growing number of publications reported the use of umbilical cord blood as the primary source for the production of platelet rich plasma (PRP) and fibrin, which are applied in clinical practices [40,41,42]. 

In this study, human peripheral blood platelet lysate (PB-PL) and human umbilical cord blood platelet lysate (CB-PL) were evaluated as possible substitutions to FBS in culture medium for human MSCs (hMSCs) culture. In order to limit the biological variability of platelet concentrations between the human donors that could result in batch to batch variation of platelet lysate pooling of the peripheral blood and cord blood units was applied for the production of PB-PL and CB-PL respectively. Thus, the probability for variations on MSCs isolation, expansion and trilineage differentiation was minimized.

In addition, an evaluation of the growth factor levels with targeted proteomic methods including MRM was performed. Finally, we tested three different additives in culture media including PB-PL, CB-PL and FBS in order to compare the isolation, expansion and tri-lineage differentiation capacities of hMSCs.

## 2. Materials and Methods

### 2.1. Preparation of Human Platelet Lysate

#### 2.1.1. Peripheral Blood Platelet Lysate

Peripheral blood units (*n* = 50) with an average volume of 450 ± 45 mL were collected from healthy donors at the Evagelismos Hospital, following the Greek regulatory procedures for blood donation. The blood units that were used for the production of hPB-PL were expired and considered as not valid for transfusion. Total platelets (PLTs) in each blood unit were determined by a haematological analyser (Nihon Khoden, MEK-6400C, Tokyo, Japan). The blood units were centrifuged at 1050× *g* for 15 min. Then, the supernatant, containing plasma and platelets, was isolated and centrifuged again at 3972× *g* for 15 min to comprise 1 unit of PRP and finally stored overnight at −80 °C. After at least 12 h of storage at −80 °C, the PRP units were thawed at 4 °C for 12 h and centrifuged at 3972× *g* for 30 min. Five PRP units were used for the production of 1 PRP pool. Each PRP pool contained about 655 × 10^6^ PLTs/mL (Table 1). The supernatants were passed through 0.65 μm filter, reducing in this way the membrane fragments, resulting in the production of platelet lysate. Finally, the PB-PLs were stored in 20-mL PL bags (Macopharma SA, Mouvaux, France) at −80 °C until further processing.

#### 2.1.2. Cord Blood Platelet Lysate

Umbilical cord blood units (*n* = 100) with an average volume of 90 ± 7 mL were collected after informed consent from the mothers by experienced midwives and immediately distributed to HCBB. The collections were performed in accordance with the ethical standards of the Greek National Ethical Committee and were approved by our Institution’s ethical board. The cord blood units were collected from end term normal and caesarean deliveries (gestational ages 36–40 weeks)—which had been processed within 24 h after collection and which did not fulfil the criteria outlined by the Hellenic Cord Blood Bank (HCBB)—for processing and storage in liquid nitrogen. A detailed description of HCBB criteria is available in supplementary Table S1. According to their blood type, cord blood units were pooled in a final volume of 400 ± 50 mL. Automated cell counting in each cord blood unit with haematological analyser was performed for determination of total cell concentration. Then, centrifugation was performed at 324× *g* for 9 min (at 22 °C optional). The supernatant was isolated and centrifuged again at 3972× *g* for 15 min to comprise 1 unit of PRP. PRP units were pooled (270 ± 30 mL) and frozen at −80 °C. Each PRP pool contained about 698 × 10^6^ PLTs/mL. Cord blood platelet lysate was prepared in accordance with the protocol described in peripheral blood platelet lysate preparation and stored at −80 °C until further processing.

### 2.2. Protein Determination and Quantification Using Multiple Reaction Monitoring

All platelet lysate samples either derived from peripheral (*n* = 4) or cord blood (*n* = 4) were centrifuged in order to remove insoluble material, prior to processing. The total protein content for each sample was determined by the Bradford assay. An appropriate volume (~2 µL) of each mixture, corresponding to 10 μg of total protein, was diluted to a volume of 20 μL with urea buffer (8 M urea, 50 mM NH_4_HCO_3_) followed by reduction (10 mM DTE) and alkylation (40 mM Iodoacetamide). The samples were then diluted to a final volume of 90 μL with 50 mM NH_4_HCO_3_ in order to obtain a final concentration of 1.5 M for urea. Trypsin was added at an enzyme protein ratio of 1:100 and the solution was incubated overnight. After trypsinization, samples were acidified with 0.1% formic acid, desalted by zip-tip and dried (speedVac). Subsequently, the samples were reconstituted in appropriate volume of mobile phase A (water, 0.1% formic acid) to a final protein concentration of 0.5 μg/μL). These samples were analysed by LC/MRM. In total, 16 growth factors were quantified with the above method. The human spectral library was searched using the Skyline software and Peptide Atlas repository to identify proteotypic peptides for the growth factors of peripheral and cord blood platelet lysate. Data analysis was performed using Skyline software and all chromatograms were manually inspected to ensure the quality and accuracy of peak picking. The sum of peak areas of two to four transitions per peptide was used to calculate the signal intensity for the selected growth factors. A detailed list of MRM transitions is available in supplementary Table S2.

### 2.3. Liquid Chromatography-Mass Spectrometry Setup

Liquid chromatography was performed using an Agilent 1200 series nano-pump system (Agilent Technologies Inc., Wilmington, DE, USA), coupled with a C18 nano-column (150 mm × 75 μm, particle size 5 μm) from Agilent. Peptide separation and elution was achieved with a 40 min 5–45% ACN/water 0.1% FA gradient at a flow rate of 300 nL/min. Six microliters of each sample (corresponding to 3 µg of total protein content) were injected.

Tryptic peptides were analysed on an AB/MDS Sciex 4000 QTRAP (AB SCIEX Pte Ltd., Orlando, FL, USA), with a nanoelectrospray ionization source controlled by Analyst 1.5 software (Sciex). The mass spectrometer was operated in MRM mode, with the first (Q1) and third quadrupole (Q3) at 0.7 unit mass resolution. Two to four transitions were recorded for each peptide. Optimum collision energies for each transition were automatically calculated by the Skyline software (v4.1, ProteoWizard, Washington, DC, USA).

### 2.4. Collection of Human Umbilical Cords

Fresh human umbilical cords (5 to 10 cm) were collected from normal deliveries (gestational ages 36–40 weeks) after informed consent form the mothers by experienced midwives trained in cord blood collection. The umbilical cords (*n* = 10) were stored into Phosphate Buffer Saline 1× (PBS 1×, Gibco, Life Technologies, Grand Island, NY, USA) supplemented with 10 U/mL Penicillin & 10 μg/mL Streptomycin (Gibco, Life Technologies, Grand Island, NY, USA) at 4 °C and processed within 24 h from reception at the HCBB. The collections were performed in accordance with the ethical standards of the Greek National Ethical Committee and were approved by our Institution’s ethical board.

### 2.5. Isolation and Culture of Wharton’s Jelly MSCs

After removing the umbilical arteries and vein, the Wharton Jelly tissue was cut with scissors into small pieces (1–3 mm^3^), placed into 6-well plates (Costar, Corning Life Sciences, Canton, MA, USA) and cultured with growth media in a humidified atmosphere with 5% CO_2_ at 37 °C. Upon reaching the sufficient number of adherent cells in the 6-well plates, cells were detached using 0.25% trypsin EDTA solution (Gibco), washed with PBS 1× and re-plated into the 75 cm^2^ flasks (Costar) with the appropriate culture medium. On reaching 80% of confluency, the cells were trypsinized, washed and resuspended into the 175 cm^2^ flasks. The same procedure was repeated until the cells reached passage 5 (P5) of culture. The growth media that were used for the expansion of Wharton’s Jelly-Mesenchymal Stromal Cells (WJ-MSCs) was α-Minimum Essentials Medium (α-MEM, Gibco) supplemented either with 15% Foetal Bovine Serum (FBS, Gibco) or 10% PB-PL or 10% CB-PL. Each growth medium was supplemented with 10 U/mL penicillin (Gibco) and 10 μg/mL streptomycin (Gibco) and 2 mM l-glutamine (Gibco). The growth medium was changed twice every week and the cultures were maintained in a humidified atmosphere with 5% CO_2_ at 37 °C.

### 2.6. Cell Viability and Growth Rate 

Cell viability of the FBS and PB-PL expanded WJ-MSCs was determined using Trypan blue. The comparison of cell doubling time (CDT) between the three different culturing conditions until reaching P5 was estimated, by plating at P1 1000 cells/cm^2^ in flasks. The number of population doubling was calculated by the classical formula:

(1) $$CDT\;=\;\frac{\log_{10}\left(N/N_{0}\right)}{\log_{10}\left(2\right)}x\;\left(T\right)$$

where *N* is the number of cells at the end of the culture, *N*_0_ is the number of cells seeded and *T* is the culture duration in hours.

### 2.7. Differentiation Capacity of MSCs

The capacity of the FBS and PB-PL expanded WJ-MSCs to differentiate into the osteogenic, chondrogenic and adipogenic lineages was determined. For this purpose, the cells were seeded in 6-well plates. WJ-MSCs were differentiated into osteogenic lineage using basal medium (Mesencult, StemCell Technologies, Vancouver, BC, Canada) supplemented with 15% osteogenic stimulatory supplements (StemCell Technologies), 0.01 mM dexamethasone (StemCell Technologies) and 50 μg/mL ascorbic acid (StemCell Technologies). Osteogenic differentiation was assessed after 25 days with Alizarin Red S (Sigma-Aldrich, Darmstadt, Germany) staining. Chondrogenic differentiation was induced in a spheroid culture using high glucose D-MEM supplemented with 0.01 mM dexamethasone (StemCell Technologies), 35 μg/mL ascorbic acid-2-phospate (StemCell Technologies), 10 ng/mL transforming growth factor- β1 (Sigma-Aldrich), liquid medium supplement (ITS+ premix, Sigma-Aldrich) for 30 days. The pellets were fixed with 10% formalin (Sigma-Aldrich), paraffin embedded and cut into 5 μm sections. Chondrogenic differentiation was assessed with Alcian blue (Fluka, Sigma-Aldrich) staining. Finally, the adipogenic differentiation of WJ-MSCs was committed with the use of basal medium (Mesencult, StemCell Technologies) supplemented with 10% of adipogenic stimulatory supplements (StemCell Technologies) for 25 days and assessed by staining of lipid vacuoles with Oil Red-O (Sigma-Aldrich) staining. 

### 2.8. Colony-Forming Unit-Fibroblast (CFU-F) Assay

The CFU-F assay performed in MSCs expanded either with FBS (*n* = 3) or PB-PL (*n* = 3) at passage 2, 3, 4 and 5. The MSCs were trypsinzed, counted and seeded at a density of 500 cells/well on 6-well tissue culture plates with MSC growth medium without the addition of FBS or PB-PL and cultured for a time period of 15 days in humidified atmosphere at 37 °C. The medium was changed biweekly. After 15 days of cultivation, cells were fixed with formalin (Sigma-Aldrich) 10% for 5 min and stained with Giemsa (Sigma-Aldrich). The stained colonies were counted manually by two independent observers.

### 2.9. Phenotypic Characterization of WJ-MSCs

Expanded WJ-MSCs with FBS (*n* = 3) and PB-PL (*n* = 3) were analysed for cell surface antigen phenotyping using flow cytometry. Each sample was measured in triplicate. Cells were labelled with fluorescein isothiocyanate-conjugated anti-CD90 (Immunotech, Beckman Coulter, Marseille, France), HLA-ABC (Immunotech), CD29 (Immunotech), CD19 (Immunotech), CD31 (Immunotech), CD45 (Immunotech). Epitopes CD105 (Immunotech), CD73 (Immunotech), CD44 (Immunotech), CD34 (Immunotech) CD3 (Immunotech) and CD14 (Immunotech), HLA-DR (Immunotech) were assessed with phycoerythrin-conjugated and PC5-conjugated mouse anti-human monoclonal antibodies respectively. The WJ-MSCs phenotypes were analysed in Cytomics FC 500 (Beckman Coulter, Marseille, France) flow cytometer with the CXP Analysis software (Beckman Coulter).

### 2.10. Growth Promotion Study and Media Validation

All peripheral blood and cord blood units used for the production of platelet lysate were tested for bacterial and viral contamination. Specifically, all blood and cord blood units were tested for aerobic and anaerobic bacteria with the BacT/Alert system for a time period of 14 days (BACTEC 9240, Becton Dickinson, Franklin Lakes, NJ, USA) by direct inoculation of at least 1% of the unit or 16 mL of the pooled platelet lysate. Further confirmation of the BacT/Alert system was performed by the use of blood and Sabouraud agar. For viral contamination, all blood and cord blood units were evaluated for HIV I/II, HBV, HGV, HTLV-I/II, CMV, HCV, HAV, WNV and for T. Pallidum and T. Cruzi, with serologic testing. In addition, final MSC culture expanded either with FBS or platelet lysates were tested for bacterial contamination, endotoxin content and mycoplasma contamination. Briefly, for sterility evaluation, 16 mL of the final cell product (MSCs cultured with FBS or platelet lysate) were tested for aerobic and anaerobic bacteria using the previously described BacT/Alert system for a time period of 14 days. The endotoxin content evaluation was performed by the Limulus amebocyte lysate (LAL) test according to European Pharmacopeia (PBI S.p.A., Milano, Italy). Finally, the MycoA-lert test (Cambrex Corporation, Verviers, Belgium) was used for the Mycoplasma contamination.

### 2.11. Statistical Analysis

Statistical analysis was performed by using Graph Pad Prism v 6.01 (GraphPad Software, San Diego, CA, USA). Comparisons in CDT and between the two experimental conditions (FBS and PB-PL) were performed with the unpaired nonparametric Mann–Whitney *U*-Test. Statistical significant difference between group values was considered when *p* value was less than 0.05. Indicated values are mean ± standard deviation.

## 3. Results

### 3.1. Preparation of Human Platelet Lysate

#### 3.1.1. Peripheral Blood

The PRP pools (*n* = 10) with an average volume of 400 ± 45 mL, were processed in order to isolate the platelet lysate. Cell counting with haematological analyser was performed prior to the freezing process. The average platelet concentration was 655 ± 21 × 10^6^/mL (Table 1). The mean platelet concentration of each peripheral blood unit is presented in supplementary Table S3.

#### 3.1.2. Cord Blood

The cord blood-derived PRP pools (*n* = 10) with an average volume of 268 ± 31 mL, were processed in order to isolate the platelet lysate. Automated cell counting in each pool was performed. The average platelet concentration was 698 ± 23 × 10^6^/mL (Table 1). The mean platelet concentration of each cord blood unit is presented in supplementary Table S3.

### 3.2. Protein Determination and Quantification in PB-PL and CB-PL

The total protein content of PB-PL and CB-PL was 88.2 ± 2.7 μg and 31.1 ± 4.3 μg respectively. The identification and quantification of growth factors in PB-PL and CB-PL was accomplished with the MRM technology. In order to obtain reliable results of the growth factor content in PB-PL and CB-PL, normalization based on the initial total protein amount of each sample was performed (PB-PL, CB-PL). The relative signal intensity of each growth factor in PB-PL and CB-PL samples is presented in supplementary figure (Figure S4). The ratio of the amount for growth factors in CB-PL in comparison to PB-PL is presented in Table 2. These results indicated that the PB-PL contained significantly elevated levels of each growth factor when compared to CB-PL.

### 3.3. Isolation and Culture Characteristics of WJ-MSCs

**Figure 1:**
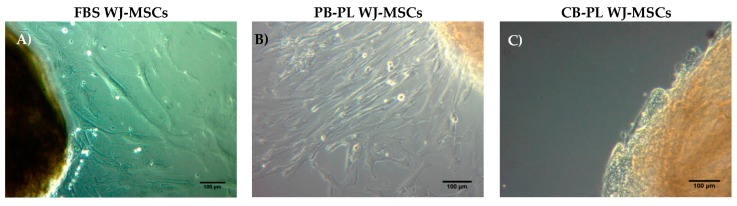
Isolation of MSCs from Wharton Jelly tissue using growth media supplemented with 15% FBS, 10% PB-PL and 10% CB-PL. (**A**) FBS expanded WJ-MSCs after 6 days of culture under standard conditions; (**B**) PB-PL expanded WJ-MSCs after 7 days of culture; (**C**) CB-PL growth medium failed to expand the WJ-MSCs even after 20 days of culture under standard conditions. Original magnification 10×, scale bars 100 μm.

**Figure 2:**
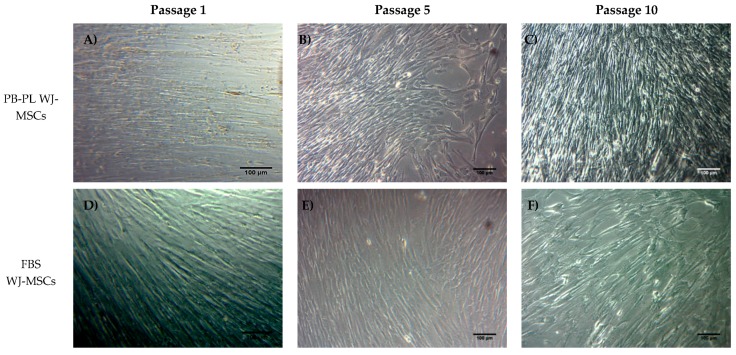
WJ-MSCs in culture at passage 1, 5 and 10. MSCs either with FBS or PB-PL growth medium achieved to retain their morphology until reaching passage 10. PB-PL expanded WJ-MSCs at passage 1 (**A**), 5 (**B**) and 10 (**C**). FBS expanded WJ-MSCs at passage 1 (**D**), 5 (**E**) and 10 (**F**). Original magnification 10×, scale bars 100 μm.

**Figure 3:**
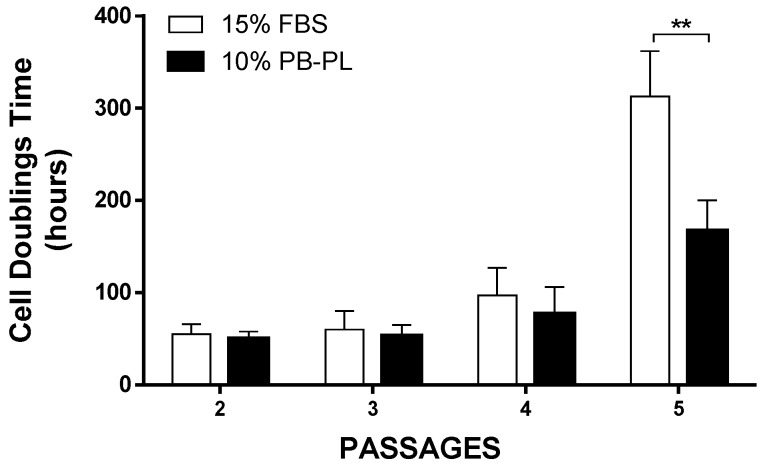
Cell doubling time of WJ-MSCs treated with FBS and PB-PL growth medium. At passage 5, the WJ-MSCs treated with PB-PL growth medium showed statistical significant reduction in CDT, compared to FBS expanded WJ-MSCs. The significance of the difference between the FBS and PB-PL treated WJ-MSCs at passage 5 is represented: ** *p* < 0.01.

WJ-MSCs were successfully isolated from 10 human umbilical cords using the growth media supplemented either with 15% FBS or 10% PB-PL. The first adherent cells appeared after 6 days of culturing using 15% FBS and after 7 days using 10% PB-PL (Figure 1) under standard conditions. The cells were passaged in 75 cm^2^ flasks after 18 days. Furthermore, there were no significant morphological differences and exhibited spindle shape morphology. However, PB-PL cells had smaller size as defined by the optical examination with the light microscope. The cells isolated from Wharton- Jelly tissue using both growth media retained their morphology until reaching P10 as shown in Figure 2. In contrast, all attempts to isolate adherent cells from human umbilical cord tissue using the CB-PL growth medium failed, even after 20 days of culture (Figure 1). Thus, only the FBS and PB-PL expanded WJ-MSCs were used for the next set of experiments for this study. The PB-PL expanded WJ-MSCs grew significantly (*p* < 0.01) faster than FBS- expanded WJ-MSCs. To calculate the doubling time of the WJ-MSCs from 10 different human umbilical cords, the cells were grown at maximum of 80% confluence until reaching P5. Our results showed longer doubling time in MSCs (313 ± 49 h) with the FBS containing medium (Table 3). The PB-PL expanded WJ-MSCs showed a higher proliferation rate as the doubling time is significantly lower at 137 ± 21 h (Figure 3).

### 3.4. Differentiation of WJ-MSCs

**Figure 4:**
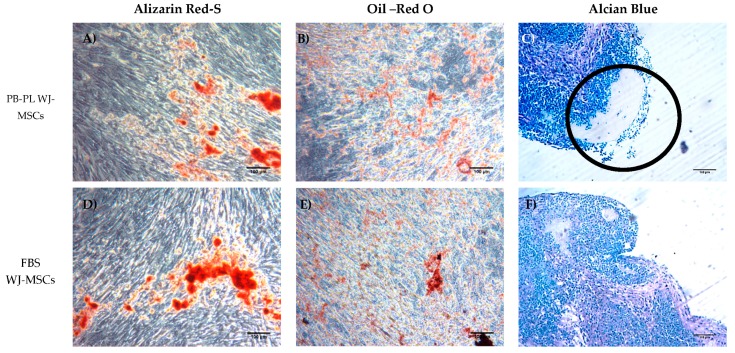
Histological analysis of the induced PB-PL and FBS treated WJ-MSCs into osteogenic, adipogenic and chondrogenic lineages. (**A**,**D**) Staining of the PB-PL and FBS expanded WJ-MSCs after induction into osteogenic lineage with Alizarin Red S stain. (**B**,**E**) Staining of the PB-PL and FBS expanded WJ-MSCs after induction into adipogenic lineage with Oil Red O stain. (**C**,**F**) Staining of the PB-PL and FBS expanded WJ-MSCs after induction into chondrogenic lineage with Alcian Blue stain. (**C**) The high amount of glycosaminoglycan content that was produced in PB-PL expanded WJ-MSCs is indicated by the black circle. Original magnification 10x, scale bares 100 μm.

The ability of WJ-MSCs to differentiate to osteogenic, chondrogenic and adipogenic lineages was analysed under particular culture conditions that favour each specific differentiation pattern. MSCs obtained at P2 were exposed to osteogenic, adipogenic and chondrogenic medium for up to 3 weeks. Both WJ-MSCs successfully exhibited calcium deposition and stained positively with Alizarin Red S stain, which is specific for calcium mineralization (Figure 4). However, the stain was more intense in PB-PL expanded cells comparing to FBS expanded cells, indicating a more robust deposition of calcium at the same time point. On the other hand, after 21 days of culturing in adipogenic conditions, both types of WJ-MSCs exhibited limited number of lipidic inclusions visualized with Oil Red-O stain, indicating an immature adipocyte phenotype. Finally, when the WJ-MSCs were induced to differentiate into chondrogenic lineage, there was a visible difference in glycosaminoglycans production between the FBS and PB-PL cultured MSCs. The glycosaminoglycan content of the PB-PL MSCs assessed was higher with Alcian blue stain comparing to the FBS expanded MSCs (Figure 4).

### 3.5. CFU–F of WJ-MSCs Cultured with FBS or PB-PL

**Figure 5:**
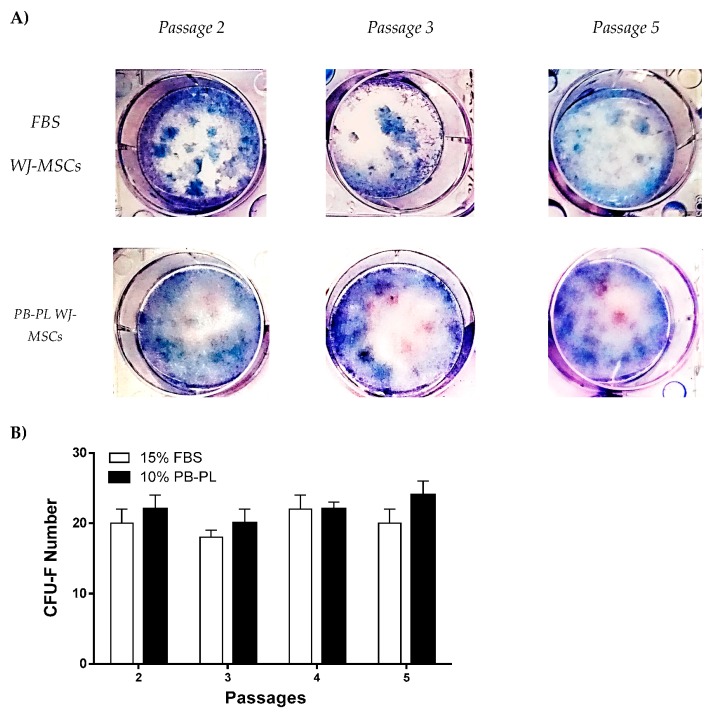
Colony Forming Unit-Fibroblast assay of FBS and PB-PL expanded WJ-MSCs. (**A**) Representable images of CFU-F at passages 2,3 and 5stained with Giemsa (**B**) The CFU-F assay was performed at passages 2,3,4 and 5 where no statistical significant difference was observed between FBS and PB-PL expanded WJ-MSCs.

The clonogenic potential of WJ-MSCs were evaluated with the CFU-F assay. More specifically, after 15 days of cultivation at 37 °C the WJ-MSCs were fixed and stained with Giemsa. The WJ-MSCs that initially isolated and expanded with culture medium containing PB-PL characterized by higher CFU-F number than the WJ-MSCs that isolated and cultured with medium containing FBS at passage 2,3,4 and 5 but this increase was not statistical significant (Figure 5). The highest CFU-F number of FBS expanded WJ-MSCs was at passage 4 (22 ± 2 CFUs) and for PB-PL was at passage 5 (24 ± 2 CFUs).

### 3.6. Phenotypic Characterization

The phenotypic characterization of WJ-MSCs was carried out with a routinely-used panel for cell surface markers as indicated by the International Society for Cellular Therapy. Three FBS expanded MSCs and three PB-PL expanded MSCs samples were analysed at P3. Both WJ-MSCs were negative to hematopoietic markers CD3, CD19, CD34 and CD45 and CD31, HLA DR. In addition, the MSCs samples were positive for β1 integrin subunit CD29 and matrix receptors CD90, CD105, CD73 and HLA-ABC. Specifically, the comparison in cell surface markers showed some minor variability in their expression between FBS and PB-PL expanded MSCs. Finally, statistical significant difference (*p* < 0.05) was noticed in positive markers CD105, CD73, CD44 and in the hematopoietic negative market CD3 between the two groups. Detailed information on the expression of cell surface markers is described in Table 4.

### 3.7. Growth Promotion Study and Media Validation Test Results

The blood and cord blood units tested for bacterial and viral contamination, were found to be negative for these pathogens. Additionally, at the end of the culture, all WJ-MSCs expanded either with FBS or PB-PL were found to be within acceptable ranges in all performed tests. Specifically, cultures were found to be negative for bacterial contamination with 14 days of cultivation and even after the use of blood and sabouraud agar no microorganism growth was observed. In regard to viral contamination, cell products were found to be within the acceptable values. Furthermore, endotoxin content of the MSC final cultures were below 2.5 EU/µ as defined by European Pharmacopoeia and mycoplasma testing results confirmed that no contamination was detected. A detailed description of the above results is presented in supplementary Tables S5 and S6. 

## 4. Discussion

The aim of this study was the evaluation of peripheral blood and cord blood platelet lysates on the isolation, expansion and differentiation of WJ-MSCs. Currently, the most widely used supplement for the in vitro expansion of MSCs is FBS [43,44,45]. Despite its great benefits such as rapid cell expansion and maintenance of tri-lineage differentiation capability, the use of FBS in cell cultures is associated with safety concerns [46,47]. Under this scope, the use of platelet lysate from blood units not valid for transfusion has been proposed and used successfully by several groups [46,47,48]. However, the availability of ready to use expired blood units is limited. On the other hand, cord blood could possibly be used for the production of platelet lysate [40,41,42]. On a daily basis, a significant number of cord blood units are rejected by the cord blood banks due to stringent selection criteria for hematopoietic stem cell isolation and cryopreservation. It is estimated that only 10–20% of cord blood units fulfilled the criteria for transplantation to patients while the remaining 80% could be used as a source of PL production. The culture media used for the isolation and expansion of WJ-MSCs in the current study, were supplemented with 15% FBS or 10% platelet lysate derived from peripheral or cord blood units.

The platelet lysate was produced from PRP pools either from peripheral blood units (*n* = 50) or cord blood units (*n* = 100) with a mean platelet concentration at 655 ± 21 × 10^6^/µ and 698 ± 22 × 10^6^/mL respectively. The production of PRP pools was performed in order to avoid batch to batch variations of peripheral blood or cord blood units. In addition, patients with severe disease conditions may be unable to donate large volumes of peripheral blood, in order to be used for autologous platelet lysate preparation. Despite this fact, the cell number that can be obtained from a single patient is very low and can be restricted further after processing steps of platelet lysate. Moreover, large scale expansion of MSCs is required for regenerative medicine applications. This huge number of MSCs can be achieved only by the use of 10–50 conventional tissue culture flasks, thus approximately 150 mL of platelet lysate it is needed for the preparation of culture media. Under this scope and in case of routinely used platelet lysate for clinical-grade expansion of MSCs under GMP, pooling of initial peripheral blood and cord blood units must be performed. As a consequence of the biological variability of platelet concentration in peripheral blood units, measurement of the platelet number in the haematological analyser of initial units has been performed. The obtained results did not show huge discrepancies between each peripheral blood or cord blood unit. Finally, after pooling and production of the PL, the platelet number measured again and found no statistical significant difference between PB and CB-PL.

Our data showed that only 15% FBS and 10% PB-PL successfully achieved the in vitro isolation of MSCs from human umbilical cord tissue after 6 days of culturing under standard conditions. In contrast, the isolation of MSCs from the Wharton Jelly tissue with the use of CB-PL growth medium was unsuccessful. Even after 20 days of culturing with bi-weekly change of the media containing 10% CB-PL, no MSCs were obtained. In this way, further evaluation of only PB-PL as replacement of FBS was performed. In addition, a targeted proteomic approach, MRM, was used for quantification of the growth factors in PB-PL and CB-PL, in order to correlate the growth factor levels with the proliferation and differentiation of the WJ-MSCs. WJ-MSCs that were expanded either with 15% FBS or 10% PB-PL growth media, retained their spindle shape morphology up to passage 10. The WJ-MSCs treated with 10% PB-PL exhibited smaller size as has been previously reported by Chevallier et al. [49], possibly due to their increased proliferative activity compared to 15% FBS treated WJ-MSCs. Furthermore, the WJ-MSCs treated with both growth media successfully differentiated to osteogenic and chondrogenic lineages as confirmed by Alizarin Red S and Alcian blue stains respectively. Moreover, when we tried to compare our study with the study of Chevallier et al. [49], it was noticed an immature adipogenic phenotype of differentiated MSCs with the presence of few lipidic inclusions observed after Oil Red O staining. The fact that these two studies did not succeed in exhibiting a mature phenotype under adipogenic differentiation conditions, might be due to the foetal origin of MSCs and the differentiation protocol that was applied [49]. Alcian blue stain revealed a higher content of glycosaminoglycans in 10% PB-PL treated WJ-MSCs rather than in 15% FBS treated WJ-MSCs. These findings were in accordance with the study of Ranzato et al. [50], who reported an increased production of collagen content after platelet lysate treatment in human non tumorigenic keratinocytes [50]. It is known that glycosaminoglycans are forming large polymers with a core protein—called proteoglycans—thus holding the collagen fibres in a specific orientation. The observed increased glycosaminoglycan and collagen amount seemed to be relevant to our study and Ranazato’s et al. [50] study, due to their common biological function and resulted by the high levels of growth factors in PB-PL. On the other hand, in regard to the osteogenic induction of MSCs, we did not notice any increased calcium deposition in 10% PB-PL treated WJ-MSCs, when compared to the previously mentioned study of Chevallier et al. [49]. In both studies, PB-PL growth medium was used from the beginning of the isolation and expansion of MSCs. A possible explanation for the different outcome in mineralization levels after osteogenic induction could be due to the different origin of MSCs that were used [49]. The origin of MSCs is of paramount importance, for the establishment of the epigenetic landmarks in their genome, suggesting that bone marrow MSCs can be driven towards the osteogenic lineage, whereas chondrocyte-like cells can be obtained easier from WJ-MSCs under differentiation conditions. Additionally, small differences might occur at the osteogenic induction protocol of the two studies, thus contributing to the final outcome [49].

As the final step for the completion of this evaluation, we investigated the proliferation, clonogenic activity and phenotypical characteristics between the two groups in this study. The use of a relatively small amount of PB-PL in the culture medium, resulted in accelerated cell growth and an increased number of cells in comparison with the FBS culture medium. This was confirmed by calculating the CDT of MSCs between passages 1 and 5. Specifically, until passage 5 the mean CDT of the PB-PL treated MSCs was 167 ± 33 h, while the mean CDT of FBS treated MSCs was 313 ± 49 h, thus strongly indicated an accelerating proliferation phenotype that was adopted by the WJ-MSCs treated with PB-PL growth medium. The cell viability in the two groups did not show any differences at passage 5 with a mean value of 85 ± 4% for the FBS expanded WJ-MSCs and 86 ± 3% for the PB-PL expanded WJ-MSCs. These positive effects on MSCs proliferation and the maintenance of tri-lineage differentiation by PB-PL were previously reported by other groups by using a lower initial percentage of PB-PL in the culture medium [47,48,49]. Regarding the clonogenic potential of WJ-MSCs, the CFU-F assay was performed. Interestingly, the MSCs cultured with PB-PL presented higher number of CFU-F when compared to FBS expanded WJ-MSCs but this increase was not statistically significant. The successful production and maintaining of CFU-F number from passage 2 to passage 5 by WJ-MSCs indicated further the preservation of their self-renewal and clonogenical properties. Furthermore, flow cytometry analysis of MSCs yielded similar results with previous studies for the expression of surface antigens [7,17]. High expression was observed for CD90, CD105 and CD73, whereas the expression of CD45, CD34, CD19, CD3, CD31, CD14 and HLA-DR was less than 2% of the isolated MSCs. Moreover, statistical significant differences were observed in CD105 and CD73 expression between FBS expanded WJ-MSCs with 97 ± 1% for CD105, 96 ± 1% for CD73 and PB-PL expanded WJ-MSCs with 99 ± 1% for CD105 and 98 ± 1% for CD73. These differences might reflect a different phenotype acquired by the WJ-MSCs upon expansion with 10% PB-PL, capable for a more robust proliferation activity and at least bilinear differentiation to osteocyte and chondrocyte- like cells.

Our results indicated altered phenotype and functionality of PB-PL treated WJ-MSCs in comparison to cells treated with the regular medium. In addition, despite our efforts no cells were isolated from Wharton Jelly tissue by using 10% CB-PL growth medium. This discrepancy could be due to the amount of growth factors presented in cord blood and peripheral blood. A number of studies have previously aimed to the identification of the exact amount of growth factors contained in platelet lysates [50,51,52,53]. The majority of these studies used ELISA assays for the quantification of platelet lysate growth factors. However, ELISA has limited multiplexing capabilities and, in order to ensure that the antibody has satisfactory specificity a Western blot assay is often required. On the other hand, MRM is currently used in preclinical and clinical studies for biomarker discovery, development and validation, thus offering a more feasible method for quantification of a panel of candidate proteins in a large number of samples [54]. In this study, MRM was the optimum method used for the quantification of growth factors, indicating the novelty of this study regarding to previous reports [50,51,52,53]. We were able to quantify 23 proteins both in peripheral blood and cord blood platelet lysate. Based on the MRM results and CB-PL/PB-PL ratio calculation, PB-PL contained higher amounts of the growth factors than CB-PL. Among them, FGF, PDGF-A, VEGF-A, TGF-b1, TNF-α, IL1α, IL-1β, IL6, IL8, the key players in proliferation stimulation and preservation of stemness identity in MSCs, were successfully quantified in PB-PL and CB-PL. The unsuccessful isolation of MSCs from Wharton Jelly tissue even after of 20 days of culturing, could be due to low levels of the above growth factors in CB-PL compared to PB-PL. The accelerated growth rate of PB-PL expanded WJ-MSCs could also be related with the presence of proinflammatory cytokines. As already described by others, we confirmed that PB-PL and CB-PL contained chemokines including CCL3, CCL4, CCL5 and the adhesion molecules ICAM1 and VCAM1 [39]. In addition, using the MRM technology, we were able to identify specific receptors for cytokines like IL1R, IL6R, IL10R1/2, for growth factors VGFR1/2, TGFR1/2 and for chemokines CCR1 as a result of platelet lysis. The adhesion molecule ICAM1 in combination with the cytokines TNF-α, IL-1α IL-1β and INF-γ play important roles in innate and adaptive immune reaction, involving transendothelial migration and T-cell mediated host defence [55]. Additionally, it has been shown that PDGF can upregulate the expression of ICAM1, thus acting as a co-stimulatory molecule and activating the HLA class II in antigen presenting cells [49,55]. Based on the current literature, IFN-γ can enhance the immunosuppressive behaviour of MSCs by up regulating the co-stimulatory molecules B7-H1 and IDO [56]. This effect could be further amplified with the combination of IL-1β and TNF-α [57]. The well characterized-immunosuppressive secreted molecule PGE2 seems to be up-regulated upon stimulation of MSCs with IL-6, TΝF-αand IFN-γ. Also, CCL2, ICAM1 and VCAM1 have a positive effect on MSCs adhesiveness, survival and proliferation in patients with acute lymphoblastic leukaemia [39,58]. 

The above results clearly indicated the successful use of PB-PL on the isolation and expansion of WJ-MSCs. However, concerns regarding the safely use of platelet lysates as an alternative supplement to FBS for cell culture medium still remain. Pathogen contamination of platelet lysates is still an important issue. More specifically, platelet lysates units are at particular risk of viral and bacterial contamination mostly by adventitious pathogens at the site of venipuncture or from donor blood transmitting agents. For this purpose and following the Greek regulatory procedures for blood donation, peripheral blood and cord blood units were tested for viral contamination serologically. In addition, sterility testing (BacT/Alert) for aerobic and anaerobic bacteria was performed, thus limiting the contamination possibility. Moreover, the MSC’s final culture from all groups was tested for bacteria contamination, evaluation of endotoxin content and mycoplasma. After all these tests, peripheral blood, cord blood units and MSCs final culture product were free of any pathogens, indicating that these producing cells under GMP conditions could theoretically be releasable for clinical use. Other pathogen reduction strategies such as the use of solvent detergent treatments, methylene blue/light, riboflavin/ultraviolet light and amotosalen/ultraviolet light (Intercept) can be applied but may affect the quality of the platelet lysate product and this was the primary reason that these approaches were not selected for the current study.

## 5. Conclusions

In conclusion, the WJ-MSCs were successfully isolated and expanded by the addition of PB-PL as supplement in growth medium. On the contrary, our efforts on isolation and expansion of WJ-MSCs with the use of CB-PL containing culture medium did not have any successful outcome. In this way, further analysis, using different protocols for the production CB-PL and its use in different types of cellular populations must be performed, in order to conclude safely if the CB-PL could serve as an alternative supplement for cell culture medium preparation. In addition, when we tried to compare the characteristics of PB-PL and FBS treated WJ-MSCs, the PB-PL treated MSCs exhibited increased growth rate, tri-lineage differentiation and preservation of the stemness identity. Comparing the growth promoting effects of WJ-MSCs under PB-PL or FBS culture media treatment, the efficacy of PB-PL in terms of MSCs viability and differentiation did not differ from those with the regular medium. Despite these facts, FBS, the most widely used supplement for the production of culture medium, currently contains xenogeneic antigens, so its use as a medium additive in cellular therapies could lead to patient adverse reactions [52].

Additionally, different Lots of FBS are characterized by variations in levels of growth factors, indicating that Lots with low growth factor concentration cannot be used as an effective supplement for maintaining the MSCs quality under good manufacturing practices. Finally, peripheral blood platelet lysate could be used as alternative additive for the production of MSCs culture medium, while the use of CB-PL is under debate, thus requiring further analysis. Both platelet lysates are devoid of any animal sera risks and hypothetically could efficiently be used for the maintenance and expansion of MSCs in large scale clinical translation studies.

## Figures and Tables

**Table 1:** Cord and peripheral PRP pools features.

Total Platelet Concentration (× 10^6^/mL)		*p*-Value
CB PRP Pools	PB PRP Pools	
698 ± 23	655 ± 21	0.17

Overview of the average PLT concentration in the cord and peripheral PRP pools. No statistically significant differences in Total Platelet concentration. Statistical significant is considered when *p* < 0.05.

**Table 2:** Ratio of growth factors in CB-PL and PB-PL.

Protein Identification	Accession Number	Ratio PB-PL/CB-PL
Interferon gamma receptor 1 precursor	INGR1_HUMAN	6.8 ± 1.2
Interleukin 1A	IL1A_HUMAN	7.0 ± 2.1
Interferon gamma precursor	IFNG_HUMAN	5.6 ± 1.0
Interleukin 1B	IL1B_HUMAN	5.2 ± 0.8
Tumour necrosis factor receptor type 1-associated DEATH domain protein	TRADD_HUMAN	5.4 ± 1.9
Intercellular adhesion molecule 1 precursor	ICAM1_HUMAN	4.4 ± 1.5
Tumour Necrosis Factor A	TNFA_HUMAN	4.3 ± 1.7
Interleukin 6	IL6_HUMAN	3.6 ± 0.6
Vascular Endothelial Growth Factor A	VEGFA_HUMAN	6.2 ± 4.1
Fibroblast Growth Factor 2	FGF2_HUMAN	3.8 ± 0.8
Platelet Derived Growth Factor A	PDGFA_HUMAN	3.9 ± 1.6
Interleukin 8	IL8_HUMAN	3.2 ± 0.6
C-C motif chemokine 3 precursor	CCL3_HUMAN	3.2 ± 0.7
Transforming Growth Factor B1 precursor	TGFB1_HUMAN	2.9 ± 0.3
C-C motif chemokine 5 precursor	CCL5_HUMAN	2.7 ± 0.3
Vascular Cell Adhesion protein 1 precursor	VCAM1_HUMAN	2.4 ± 0.3

**Table 3:** Cell culture kinetics of FBS and PB-PL expanded WJ-MSCs.

FBS Expanded WJ-MSCs				
	*n* = 10	*n* = 10	*n* = 10	*n* = 10
Passage	2	3	4	5
Mean cell Viability (%)	83 ± 1 ^†^	88 ± 2	87 ± 3	85 ± 4
Cell Doubling Time (hours)	55 ± 11	60 ± 20	97 ± 30	313 ± 49 ^‡^
**PB-PL Expanded WJ-MSCs**				
	*n* = 10	*n* = 10	*n* = 10	*n* = 10
Passage	2	3	4	5
Mean cell Viability (%)	88 ± 2 ^†^	86 ± 1	88 ± 2	86 ± 3
Cell Doubling Time (hours)	50 ± 8	53 ± 12	77 ± 29	167 ± 33 ^‡^

Overview of the culture kinetics of FBS and PB-PL expanded WJ-MSCs. There was no statistically significant difference between two groups, with the exception of FBS expanded WJ-MSCs CDT at P5 that was calculated higher in respect to PB-PL expanded WJ-MSCs. Additionally, the Mean Cell Viability (%) FBS expanded WJ-MSCs at P2 was lower than PB PL expanded WJ-MSCs. ^†^ *p* = 0.0008; ^‡^ *p* = 0.0001, *p* < 0.05 indicates statistical significance.

**Table 4:** Cell surface markers expression (%) at FBS and PB-PL expanded WJ-MSCs.

Cell Surface Markers	FBS Expanded WJ-MSCs	PB-Expanded WJ-MSCs	*p* Value
CD90	96.2 ± 0.6	96.7 ± 0.4	0.3707
CD105	96.8 ± 0.1	98.6 ± 0.5	0.0053
HLA-ABC	94.0 ± 0.1	94.7 ± 0.5	0.1927
CD73	96.4 ± 0.7	98.7 ± 0.6	0.0300
CD29	95.6 ± 0.7	94.6 ± 0.5	0.1784
CD44	96.0 ± 0.6	93.7 ± 0.3	0.0176
CD19	1.2 ± 0.1	1.4 ± 0.1	0.1295
CD3	1.8 ± 0.1	1.6 ± 0.1	0.0066
CD31	1.6 ± 0.2	1.6 ± 0.1	0.8620
CD14	1.3 ± 0.1	1.7 ± 0.2	0.1159
HLA-DR	1.1 ± 0.1	1.4 ± 0.2	0.1561
CD45	1.4 ± 0.3	1.3 ± 0.1	0.7445
CD34	1.5 ± 0.1	1.6 ± 0.2	0.4423

Percentage of all WJ-MSCs expressing surface markers as determined by flow cytometry. The percentage of expression is indicated as the mean of all WJ-MSCs (*n* = 3) of each group.

## Supplementary Materials

Supplementary materials are available online at http://www.mdpi.com/2306-5354/5/1/19/s1.

Supplementary File 1
